# Supplementary material for: Generation of Multiple Arbovirus-like Particles Using a Rapid Recombinant Vaccinia Virus Expression Platform
Source: Pathogens. 2022 Dec 9;11(12):1505. doi: 10.3390/pathogens11121505 (PMC9785247; doi:10.3390/pathogens11121505)
Supplement: Supplementary file 1 [file pathogens-11-01505-s001.zip › pathogens-2068569-supplementary.pdf]

Supplementary Materials

Supplementary Table S1. Cloning and sequencing primers for POWV constructs

Purpose	Sequence (5′ → 3′)
vIND-POWV (prototype lineage) PCR amplification, forward	TCTCAATGGCTAAGGCGTC
vIND-POWV (prototype lineage) PCR amplification, reverse	AGATCCAGCTTGCCTTCCAC
vIND-POWV (prototype lineage) sequencing	ACGTCAACGGGTTCTTCCTC
vIND-POWV (DTV lineage) PCR amplification, forward	TCTCAATGGCTAAGGCGTC
vIND-POWV (DTV lineage) PCR amplification, reverse	CGGGATGACCACAGACCTTC
vIND-POWV (DTV lineage) sequencing	ACACCATTCTCCCATGTCCG

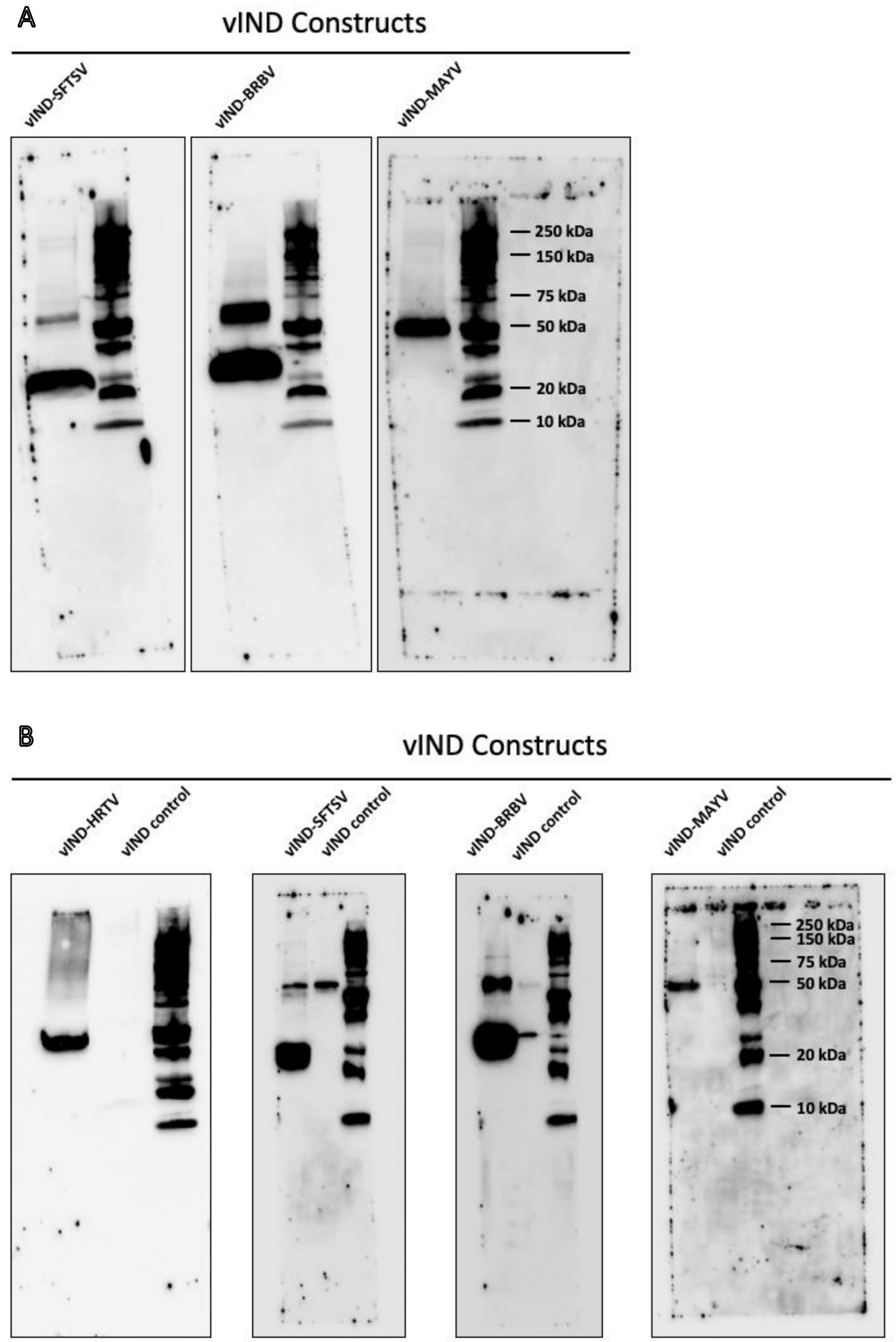

Supplementary Figure S1. Detection of protein expression (vIND-HRTV, vIND-SFTSV, vIND-BRBV, and vIND-MAYV) in western blots. PEG precipitated VLP samples were harvested from Vero cells infected with each vIND construct. A vIND that does not encode any gene of interest was used to infect Vero cells to generate a negative control. The negative control was PEG precipitated in the same manner as the test vINDs. (A) Western blots without a negative control. (B) Western blots with a negative control included.

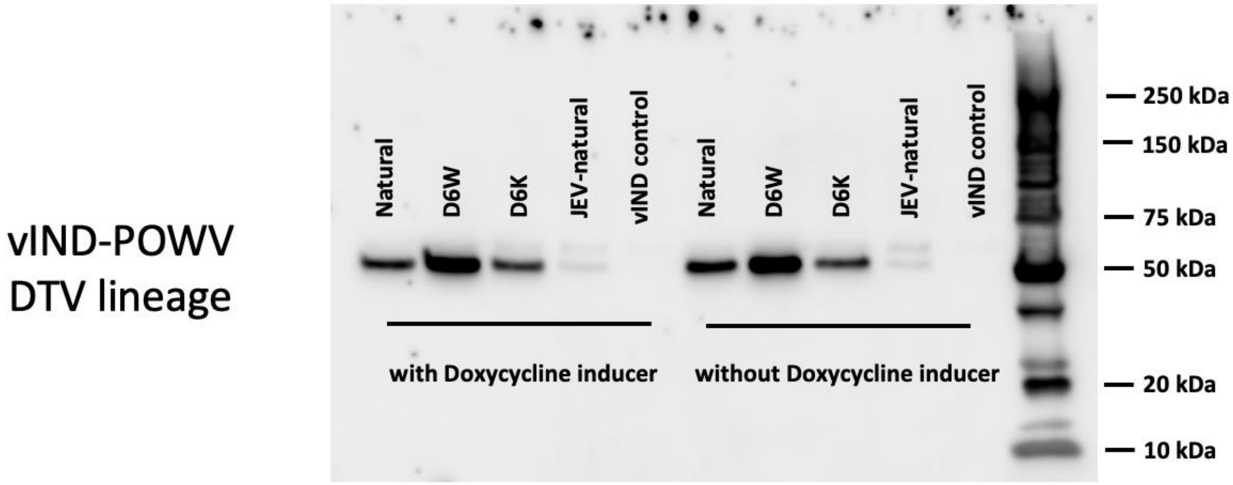

Supplementary Figure S2. Detection and comparison of protein expression (vIND-POWV) in western blots. PEG precipitated VLP samples were harvested from Vero cells infected with each vIND construct. A vIND that does not encode any gene of interest was used to infect Vero cells to generate a negative control. The negative control was PEG precipitated in the same manner as the test vINDs. Whole protein concentration of each POWV VLP sample was quantitated by Qubit 4 fluorometer (Invitrogen) to ensure equal loading amount. Two sets of Vero cells were infected, with one set in the presence of doxycycline inducer, thus allowing replication of the vINDs.

vIND-ZIKV

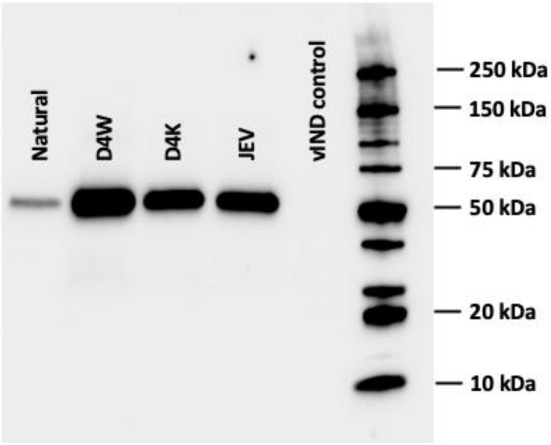

Supplementary Figure S3. Detection and comparison of protein expression (vIND-ZIKV) in western blots. Detailed information of ZIKV genes, VLP production, and western blot conditions can be found in our previous study [30]. PEG precipitated VLP samples were harvested from Vero cells infected with each vIND construct in the absence of doxycycline inducer. A vIND that does not encode ZIKV genes was used to infect Vero cells to generate a negative control. The negative control was PEG precipitated in the same manner as the test vINDs. TGX stain-free gel (Bio-Rad) was imaged before membrane transfer to ensure equal amount of protein was loaded to each well.
